# Contemporary and Emerging Therapeutics in Cardiovascular-Kidney-Metabolic (CKM) Syndrome: In Memory of Professor Akira Endo

**DOI:** 10.3390/biomedicines13092192

**Published:** 2025-09-08

**Authors:** Inderjeet Singh Bharaj, Ajit Brar, Aayushi Kacheria, Karen Purewal, Austin Simister, Umabalan Thirupathy, Palak Gupta, Jasraj Kahlon, Juzer Munaim, Ei Ei Thwe, Samer Ibrahim, Valerie Martinez Vargas, Krishnaswami Vijayaraghavan

**Affiliations:** 1 Abrazo Health Network Internal Medicine Residency Program, Glendale, AZ 85308, USA; aayushikacheria@gmail.com (A.K.); purewalkaren@gmail.com (K.P.); simisteraustin@gmail.com (A.S.); jasraj.s.kahlon@gmail.com (J.K.); juzer5272@gmail.com (J.M.); 2 Michigan State University Internal Medicine Residency Program, Hurley Medical Center, Flint, MI 48503, USA; ajitbrar466@gmail.com; 3 Cheshire Medical Center, Keene, NH 03431, USA; t_umabalan@hotmail.com; 4 Midwestern University Internal Medicine Residency Program, Verde Valley Medical Center, Cottonwood, AZ 86326, USA; palakg371@gmail.com; 5 Abrazo Health Network Cardiovascular Disease Fellowship Program, Phoenix, AZ 85016, USA; eieithwe90@gmail.com (E.E.T.); heydrsam@gmail.com (S.I.); 6 Endocrine, Diabetes and Metabolism Fellowship Program, University of Arizona, Tucson, AZ 85719, USA; valerie.mar.18@gmail.com; 7 Arizona College of Medicine, University of Arizona, Phoenix, AZ 85004, USA

**Keywords:** CKM syndrome, SGLT2 inhibitors, GLP-1 receptor agonists, finerenone, MASLD, RNA-based therapies, artificial intelligence, cardiovascular disease, chronic kidney disease, metabolic syndrome

## Abstract

Cardiovascular-kidney-metabolic (CKM) syndrome is a multifaceted, systemic disorder characterized by the interplay of cardiovascular disease (CVD), chronic kidney disease (CKD), type 2 diabetes mellitus (T2DM), and obesity. This review synthesizes current and emerging therapeutic strategies aimed at addressing the shared pathophysiologic mechanisms driving CKM progression, such as insulin resistance, inflammation, oxidative stress, and neurohormonal activation. Established pharmacotherapies that include sodium-glucose cotransporter 2 (SGLT2) inhibitors, glucagon-like peptide-1 receptor agonists (GLP-1 RAs), and nonsteroidal mineralocorticoid receptor antagonists like finerenone have demonstrated robust efficacy in reducing cardiovascular events, slowing renal decline, and improving metabolic outcomes. Additionally, novel agents targeting lipoprotein(a), interleukin-6, and hepatic fat accumulation are expanding the therapeutic landscape. RNA-based therapies, including antisense oligonucleotides (ASOs) and small interfering RNAs (siRNAs), are designed to modulate lipoprotein(a) and PCSK9 expression. Artificial intelligence (AI) is also emerging as a transformative tool for personalized CKM management, enhancing risk prediction and clinical decision-making. The review highlights the relevance of metabolic dysfunction-associated steatotic liver disease (MASLD) as a CKM modifier and discusses the approval of resmetirom, a selective thyroid hormone receptor β agonist, for noncirrhotic MASH. By integrating evidence from clinical trials, mechanistic studies, and emerging technologies, this review provides a comprehensive resource for clinicians and researchers navigating the evolving field of CKM syndrome.

## 1. Introduction

Cardiovascular-kidney-metabolic (CKM) syndrome is defined by the presence of at least two of the following conditions: cardiovascular disease (CVD), chronic kidney disease (CKD), type 2 diabetes mellitus (T2DM), or obesity. This syndrome reflects the systemic nature of these disorders which exacerbate each other, leading to increased morbidity and mortality rates, particularly from cardiovascular causes. The advisory from the American Heart Association (AHA) defines CKM syndrome as a spectrum of diseases driven primarily by excessive or dysfunctional adiposity, which significantly contributes to the progression of both CVD and CKD [1]. The relationship among these conditions is further clarified by understanding the concepts of cardiorenal syndrome, which highlights the bidirectional interplay between the heart and kidneys, and cardiometabolic syndrome, where excess body fat is linked to systemic inflammation and insulin resistance, which are both critical to the development of cardiovascular risks [2]. Metabolic dysfunction-associated steatotic liver disease (MASLD), formerly known as nonalcoholic fatty liver disease (NAFLD), and its progressive form, metabolic dysfunction-associated steatohepatitis (MASH), are not formally included in CKM syndrome definitions. However, accumulating evidence suggests that hepatic metabolic dysfunction contributes to systemic inflammation, insulin resistance, and atherogenesis, thereby exacerbating CKM-related outcomes [3]. The rising incidence of obesity and metabolic disorders has exacerbated this crisis, highlighting the necessity for a comprehensive understanding of CKM disease and the therapeutic approaches to manage it effectively [4].

The incidence of CKM syndrome remains inadequately characterized due to the lack of comprehensive data across different demographics and regions. Studies indicate that the prevalence of advanced CKM stages significantly increases with age, particularly in individuals aged 60 and above, who exhibit a prevalence ratio of 38.71 compared to younger adults aged 35 to 44 years (*p* < 0.001) [5,6]. In a study conducted by Chinese authors analyzing data from a UK registry, the prevalence of CKM syndrome among adults, including those in both early and advanced stages, rose from 77.1% in 2010 to 83.7% in 2019 [7]. This increase is consistent with global trends, as the burden of cardiovascular diseases (CVDs) has escalated significantly, with global mortality from CVDs increasing from 12.4 million in 1990 to 19.8 million in 2022 [1]. In the United States, the prevalence of CKM syndrome varies by state, with West Virginia reporting the highest prevalence at nearly 87% in 2023. Delaware follows closely, with rates rising from 76% to 83% over the same period. Generally, states in the midwestern and southern regions exhibit the highest prevalence rates, ranging from 71.8% in Colorado to 86.7% in West Virginia [8]. Men are also found to be more susceptible to advanced stages of the syndrome than women, while socioeconomic disparities affect its manifestation across different ethnic groups [9,10]. For instance, research indicates that individuals from South Asian backgrounds may have a higher likelihood of developing CKM syndrome due to genetic predispositions, combined with adverse social factors [7]. Moreover, it is crucial to recognize that socioeconomic factors play a significant role in the prevalence of CKM syndrome. Individuals with low socioeconomic status (SES) are at a higher risk of developing CVD, a key component of CKM [11]. The increased prevalence of CKM syndrome is also exacerbated by a higher occurrence of chronic diseases such as CKD and diabetes, affecting approximately 850 million and 537 million individuals worldwide, respectively [12,13].

The pathophysiology of CKM disease is rooted in a complex network of shared biological mechanisms that drive progressive dysfunction across organ systems. Central to this interplay are shared metabolic insults such as insulin resistance, hyperglycemia, oxidative stress, chronic inflammation, and neurohormonal activation [14]. These mechanisms initiate and reinforce a cycle of progressive organ dysfunction. CKD exacerbates hypertension and dyslipidemia, which in turn accelerates cardiovascular disease [15]. Similarly, heart failure impairs renal perfusion, worsening kidney function and metabolic control [16]. Addressing these overlapping pathways requires a unified therapeutic approach. Recent pharmacologic advances have significantly reshaped the treatment landscape. Agents such as sodium-glucose cotransporter 2 (SGLT2) inhibitors, glucagon-like peptide-1 receptor agonists (GLP-1 RAs), and non-steroidal mineralocorticoid receptor antagonists (e.g., finerenone) have demonstrated efficacy in slowing disease progression and reducing cardiovascular risk across diverse patient populations. Although thyroid hormone receptor β (TRβ) agonists have not yet demonstrated direct effects on CKM endpoints, their potential to modulate hepatic metabolism and systemic cardiometabolic risk warrants inclusion in this discussion.

This review is dedicated to Professor Endo, whose pioneering contributions to nephrology, cardiovascular pathophysiology, and pharmacology have profoundly shaped the field. His seminal work on renal hemodynamics, acid-base regulation, and lipid-lowering therapies laid foundational principles that continue to inform contemporary understanding of cardiorenal interactions and CKM syndrome. His influence extends beyond research: as a mentor and intellectual guide, Professor Endo has shaped the careers of countless physicians, including the authors of this review. His ability to translate complex mechanisms into clinically meaningful insights remains a model for rigorous and compassionate scholarship. We honor his enduring impact on our work and on the broader scientific community.

This review offers a timely and integrative perspective on CKM syndrome by bridging established pharmacologic therapies with emerging therapeutic targets and modifiers. Our work emphasizes the interconnected pathophysiology and the need for unified treatment strategies. We also highlight novel agents under investigation including RNA-based therapies, interleukin-6 inhibitors, and explore the potential of artificial intelligence to personalize CKM management. By synthesizing current evidence and identifying future directions, this review aims to serve as a comprehensive resource for clinicians and researchers navigating the evolving landscape of CKM care [Table 1]. We believe this contribution is both timely and necessary to advance clinical understanding and therapeutic innovation in a syndrome that is rapidly becoming a global health priority.

## 2. Materials and Methods

A comprehensive narrative literature review was conducted to evaluate the pathogenesis of CKM syndrome using major databases, including PubMed (accessed on 10 April 2025), Scopus (accessed on 10 April 2025), and Google Scholar (accessed on 10 April 2025). The search employed a combination of keywords such as “Metabolic associated steatotic liver disease”, “non fibrotic Metabolic associated steatohepatitis”, “Nonalcoholic fatty liver disease”, “Nonalcoholic steatohepatitis”, “Metabolic syndrome”, “Cardiovascular disease”, “Heart failure”, “Cardiorenal syndrome”, “Cardiometabolic syndrome”, “Chronic kidney disease”, “antifibrotic therapies”, and “goal directed medical therapy”, along with Boolean operators (AND, OR) and Medical Subject Headings (MeSH) terms to refine results and ensure relevance.

Reference management was performed using Zotero (Version 6.0.27; https://www.zotero.org, accessed on 10 April 2025). Manuscript drafting and formatting were carried out using Google Docs (https://docs.google.com, accessed on 10 April 2025). Figures were created using BioRender (https://biorender.com, accessed on 10 April 2025).

Inclusion criteria encompassed studies, review articles, and meta-analyses focusing on the relationship between MASLD, MASH, NASH, NAFLD, Metabolic syndrome, CVD, CKD, diagnostic approaches, treatment modalities, and articles exploring the pathophysiological mechanisms linking cardiorenal and metabolic syndrome. Exclusion criteria included non-English publications, studies unrelated to metabolic syndrome, studies focusing solely on non-cardiac manifestations of metabolic syndrome and chronic kidney disease, conference abstracts, editorials, and opinion pieces.

The study selection process involved title and abstract screening to assess relevance, followed by full-text review to confirm alignment with study objectives. Data were systematically extracted using a predefined framework, and findings were summarized qualitatively, focusing on key themes such as pathophysiology, the impact of CKM syndrome, morbidity and mortality, therapeutic targets, and emerging research gaps and opportunities.

## 3. Current and Emerging Therapeutics

Current and emerging therapeutics for CKM syndrome target the complex interplay between cardiovascular, renal, and metabolic disorders. Established treatments include SGLT2 inhibitors, GLP-1RAs, and mineralocorticoid receptor antagonists like finerenone, which have demonstrated benefits in reducing cardiovascular events, slowing CKD progression, and improving metabolic parameters. Emerging therapies focus on novel targets such as lipoprotein(a), inflammatory pathways, and hepatic fat accumulation. RNA-based therapies like antisense oligonucleotides and small interfering RNAs show promise in modulating gene expression related to lipid metabolism. Additionally, artificial intelligence is increasingly being leveraged to enhance risk prediction, treatment selection, and personalized management strategies in CKM syndrome.

### 3.1. Sodium Glucose Co-Transport 2 Inhibitors

SGLT-2 inhibitors were originally developed for the management of T2DM. However, their use has now expanded into the cardiorenal and metabolic fields. This expansion is due to the growing recognition of the interconnections between T2DM, CVD, and CKD. SGLT2 inhibitors provide cardiorenal protection through multiple interrelated mechanisms that extend beyond glycemic control. The primary renal mechanism is the restoration of tubuloglomerular feedback via increased sodium delivery to the macula densa, leading to afferent arteriolar vasoconstriction, reduced intraglomerular pressure, and the mitigation of hyperfiltration injury [24,25]. This effect is central to nephroprotection and is consistently highlighted in both clinical trials and mechanistic studies [26]. SGLT2 inhibitors also induce osmotic diuresis and natriuresis, resulting in plasma volume contraction, lower blood pressure, and reduced cardiac preload and afterload, which are key contributors to heart failure benefit [27]. Anti-inflammatory, antifibrotic, and antioxidative effects—mediated by reduced macrophage activation, decreased pro-inflammatory cytokines, and improved mitochondrial function—further contribute to both cardiac and renal protection [28,29].

Initial studies evaluating cardiovascular outcomes include DAPA-HF which found dapagliflozin resulted in a 26% reduction in the composite endpoint of cardiovascular death or heart failure hospitalization compared to placebo [30]. The EMPEROR-reduced trial further exemplified the benefits of SGLT2 inhibitors as there was a 25% lower combined risk of cardiovascular death or heart failure hospitalization in patients receiving empagliflozin vs. placebo [31]. The AHA and the American College of Cardiology (ACC) both recognize previously mentioned multifactorial mechanisms as the basis for the cardiorenal benefits observed in large outcome trials [32]. In fact, SGLT2 inhibitors are now an essential component of guideline-directed medical therapy (GDMT) in the treatment of heart failure with reduced ejection fraction (HFrEF) [33]. The EMPEROR-preserved trial found that empagliflozin reduced the risk of the primary endpoint of cardiovascular death or hospitalization for heart failure by 21% in patients with heart failure and a preserved ejection fraction (HFpEF) [34]. This finding was consistent amongst generally all subgroups, including those with or without diabetes [34]. The DELIVER trial demonstrated that dapagliflozin significantly reduced the risk of worsening heart failure or cardiovascular death in patients with heart failure and a mildly reduced or preserved ejection fraction, regardless of diabetes status or baseline ejection fraction [19]. The treatment also improved symptom burden and showed a favorable safety profile, supporting its broader use across the heart failure spectrum. Ultimately, these findings have allowed for SGLT2 inhibitors to become a key player in the treatment of HFrEF/HFpEF.

Albuminuria is recognized as an independent risk factor for cardiovascular events, progression to kidney failure, and all-cause mortality [35]. A meta-analysis of 11 randomized trials evaluating SGLT2 inhibitors demonstrated a significant reduction in major adverse cardiovascular events (MACE), with the observed decrease in cardiovascular mortality primarily driven by reductions in heart failure-related deaths and sudden cardiac death. Importantly, this cardiovascular benefit was most pronounced in patients with albuminuria, who exhibited approximately twice the event rate compared to those without albuminuria [36]. The CREDENCE trial specifically assessed the efficacy of canagliflozin in patients with diabetic chronic kidney disease (CKD), revealing a 30% reduction in the risk of kidney failure [35]. Subsequent studies expanded the therapeutic role of SGLT2 inhibitors to populations without diabetes and with more advanced CKD. The DAPA-CKD trial demonstrated that, when compared to placebo, dapagliflozin significantly reduced the risk of a composite renal endpoint, including sustained decline in estimated glomerular filtration rate (eGFR) of ≥50%, end-stage kidney disease, or death from renal or cardiovascular causes independent of diabetes status [37]. Notably, 14.5% of participants in DAPA-CKD had an eGFR < 30 mL/min/1.73 m^2^, in contrast to the CREDENCE trial, which excluded patients below this threshold [35,37]. The EMPA-KIDNEY trial further reinforced the efficacy of SGLT2 inhibitors in advanced CKD, enrolling patients with eGFR as low as 20 mL/min/1.73 m^2^ [22]. This study included a broad spectrum of albuminuria, with 48.3% of participants having a urinary albumin-to-creatinine ratio (UACR) < 300 mg/g, and still demonstrated a significant reduction in CKD progression compared to placebo [22]. Reflecting these findings, the 2022 consensus report from the American Diabetes Association (ADA) and the European Association for the Study of Diabetes (EASD) endorsed the use of SGLT2 inhibitors not only for heart failure management but also for reducing MACE and improving renal outcomes [38]. Additionally, the European Society of Cardiology (ESC) has incorporated SGLT2 inhibitors into its heart failure and cardiovascular risk reduction guidelines, further reinforcing their role across both diabetic and non-diabetic populations [39].

**Figure 1:**
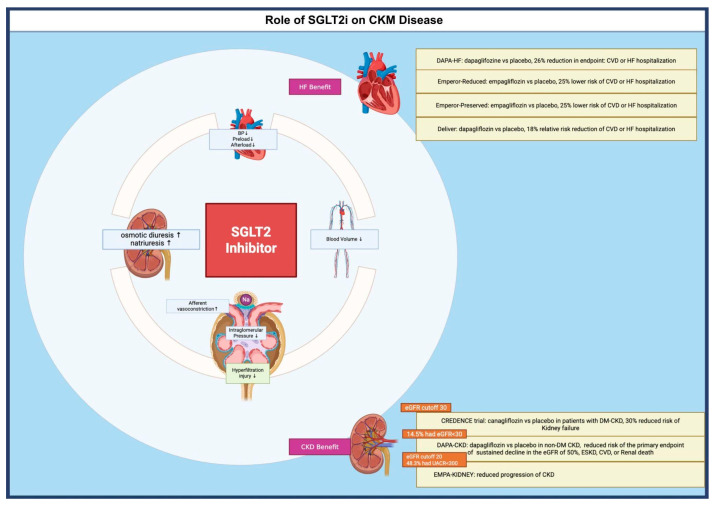
Evidence Supporting SGLT-2 Inhibitors in the Cardiovascular-Kidney-Metabolic syndrome. Legend: BP: Blood Pressure; CKD: Chronic Kidney Disease; CVD: Cardiovascular Death; eGFR: estimated glomerular filtration rate; HF: Heart Failure; ESKD: End stage kidney disease; MACE: Major adverse cardiovascular events; T2DM: Type 2 Diabetes Mellitus; and UACR: Urine Albumin Creatinine Ratio.

In summary, SGLT2 inhibitors are a cornerstone therapy for CKM syndrome, providing integrated cardiovascular, renal, and metabolic protection as endorsed by the AHA, ADA, EASD, ESC, and KDIGO. Their benefits extend beyond glycemic control and include reduction in progression of CKD, hospitalization for heart failure, major adverse cardiovascular events, and all-cause mortality, with efficacy demonstrated in patients both with and without diabetes [Figure 1].

### 3.2. Glucagon-like-Peptide-1 Receptor Agonists

GLP-1 RAs play a central role in the management of CKM syndrome, particularly in patients with T2DM, obesity, and/or CKD. GLP-1RA was initially developed for treatment of T2DM. ADA and EASD recommend GLP-1 RAs as a preferred first injectable therapy before insulin in most patients with T2DM who require intensification beyond oral agents, due to their efficacy in lowering HbA1c, low risk of hypoglycemia, and favorable effects on weight [38,40]. These pharmacological agents effectively manage glycemic levels, facilitate weight reduction, and have been shown to substantially decrease MACE and all-cause mortality in high-risk cohorts, particularly individuals with CKD and established atherosclerotic cardiovascular disease (ASCVD) [41,42]. In fact, clinical guidelines highlight the substantial cardiovascular benefits of GLP-1Ras, demonstrating a robust capacity to reduce MACE and all-cause mortality, making them a key consideration in managing this vulnerable population [38,40].

GLP-1 RAs are recommended as adjunctive therapy for adults with T2DM, who are overweight or obese, and MASLD, particularly when MASH or high risk for liver fibrosis is present [43]. Multiple phase II and phase III trials have highlighted the efficacy of GLP-1RAs in reducing hepatic fat content and liver histological inflammation and fibrosis among MASLD patients [44,45,46]. Mechanistically, GLP-1 RAs exert their hepatic benefits primarily through indirect pathways, including weight reduction, improved insulin sensitivity, and decreased systemic inflammation, rather than direct action on hepatocytes. These agents also improve cardiovascular and renal outcomes, which is particularly relevant given the high cardiometabolic risk in MASLD/MASH [47,48]. Despite these advances, no GLP-1 RA is currently approved specifically for MASLD or MASH, and regulatory approval is pending further outcome data.

GLP-1RAs are now approved as adjunctive therapy in adults with T2DM and CKD to improve glycemic control and potentially slow progression of kidney disease, with preference for agents with demonstrated cardiorenal benefit (liraglutide, semaglutide, and dulaglutide) [42]. Beyond glycemic control, GLP-1RAs exert anti-inflammatory and antioxidant effects that mitigate cellular injury and oxidative stress. Their natriuretic properties facilitate sodium excretion, contributing to improved fluid balance and blood pressure regulation [49]. Mechanistically, GLP-1RAs have been shown to reduce glomerular hyperfiltration and downregulate the receptors for advanced glycation end products (RAGE), thereby attenuating inflammation driven by glycation-mediated signaling [50]. Clinical trials have demonstrated that GLP-1RAs reduce albuminuria and slow the decline in eGFR, with efficacy observed even in patients with impaired kidney function (eGFR < 60 mL/min/1.73 m^2^) [42]. In CKD populations, these agents significantly lower the risk of composite renal endpoints, including progression to macroalbuminuria, sustained eGFR decline, kidney failure, and death from renal causes. A meta-analysis of randomized controlled trials further supports these findings, revealing consistent improvements in renal and cardiovascular outcomes, as well as enhanced survival [51]. Taken together, the multifaceted mechanisms and clinical benefits of GLP-1RAs position them as promising therapeutic options for patients with CKD, offering both direct renoprotective effects and cardiovascular risk reduction.

The American Gastroenterological Association (AGA) recommends glucagon-like peptide-1 receptor agonists (GLP-1 RAs), particularly semaglutide and liraglutide, as pharmacologic options for the management of obesity [52]. These agents promote weight loss primarily through central appetite suppression, delayed gastric emptying, and modulation of energy intake, while also offering favorable effects on glycemic control, blood pressure, and lipid metabolism [53]. Building on these guidelines, extensive clinical trial and meta-analytic evidence underscores the efficacy of GLP-1 RAs in achieving significant weight reduction among adults with obesity, irrespective of diabetes status. Randomized controlled trials and systematic reviews consistently demonstrate that semaglutide (2.4 mg once weekly, subcutaneous) and liraglutide (3.0 mg once daily, subcutaneous) yield clinically meaningful weight loss. Semaglutide has been associated with average reductions of 9–16% in body weight over one year, while liraglutide achieves reductions of 4–7%, with a substantial proportion of patients attaining ≥10% weight loss. These outcomes surpass those achieved with first-generation anti-obesity medications and lifestyle interventions alone, positioning GLP-1 RAs as highly effective agents in the contemporary pharmacologic management of obesity [54].

**Figure 2:**
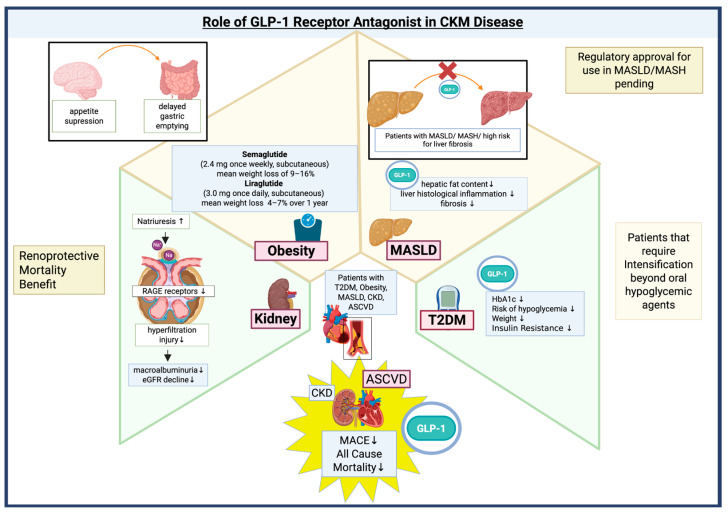
Evidence Supporting GLP-1RA in the Cardiovascular-Kidney-Metabolic syndrome. Legend: ASCVD: atherosclerotic cardiovascular disease; CKD: Chronic Kidney Disease; CVD: Cardiovascular Death; MACE: Major adverse cardiovascular events; MASLD: metabolic dysfunction–associated steatotic liver disease; MASH: metabolic dysfunction–associated steatohepatitis; Na: Sodium; RAGE: Receptor for advance glycation end products; and T2DM: Type 2 Diabetes Mellitus.

In summary, GLP-1 receptor agonists have emerged as cornerstone pharmacologic agents in the management of CKM syndrome, particularly when weight reduction, glycemic control, and ASCVD risk reduction are prioritized. Their robust efficacy in promoting sustained weight loss, improving metabolic parameters, and delivering cardiovascular benefit is well-supported by high-quality clinical trials and endorsed by leading professional societies. As such, GLP-1 RAs represent a preferred therapeutic strategy in both obesity and CKM management, aligning evidence-based outcomes with comprehensive patient care [38,55,56] [Figure 2].

### 3.3. Mineralocorticoid Receptor Antagonists

Finerenone is a nonsteroidal, highly selective antagonist of the mineralocorticoid receptor (MR), functioning by competitively inhibiting the binding of aldosterone and cortisol, thereby suppressing MR-mediated gene transcription [57]. Finerenone exhibits high potency and selectivity for the MR, with minimal affinity for androgen, progesterone, estrogen, or glucocorticoid receptors, distinguishing it from steroidal MRAs such as spironolactone and eplerenone. Compared to these agents, finerenone demonstrates a more balanced tissue distribution between the heart and kidney and exerts more potent anti-inflammatory and anti-fibrotic effects, with a lower risk of hyperkalemia and hormonal side effects [58]. This blockade occurs in both epithelial tissues (such as the kidney, where it reduces sodium reabsorption) and non-epithelial tissues (including the heart and vasculature), leading to reduced inflammation and fibrosis which are the key drivers of cardiorenal disease progression in CKD and T2DM [59].

Pathologic overactivation of the MR contributes to end-organ damage through mechanisms beyond sodium retention and hypertension, notably by promoting inflammation and fibrosis [60]. While steroidal MRAs have demonstrated clinical benefit in heart failure with reduced ejection fraction (HFrEF), their use in patients with advanced CKD has been constrained by adverse effects such as hyperkalemia and acute kidney injury, as outlined in the KDIGO guidelines [60]. Although the overall incidence of hyperkalemia with MRAs is approximately 10%, AHA recommends vigilant monitoring of serum potassium and renal function, particularly in patients with impaired kidney function [61]. Meta-analyses confirm that spironolactone and eplerenone reduce proteinuria and cardiovascular events in CKD, but both agents carry a heightened risk of hyperkalemia, with spironolactone additionally associated with gynecomastia [62]. Eplerenone is more selective and associated with fewer hormonal side effects, but both agents require careful potassium and renal function monitoring [63]. Finerenone’s selective MR antagonism provides a more targeted therapeutic approach, potentially minimizing adverse effects while preserving efficacy [58]. Data from the ARTS trial (Mineralocorticoid Receptor Antagonist Tolerability Study) support this safety profile, demonstrating a lower incidence of hyperkalemia with finerenone (4.5%) compared to spironolactone (11.1%) in patients with CKD and heart failure [64].

Several pivotal trials have demonstrated the cardiovascular and renal benefits of finerenone in patients with T2DM and CKD. The FIDELIO-DKD trial showed, compared to placebo, that finerenone significantly reduced the risk of kidney failure, sustained eGFR decline ≥ 40%, and death from renal causes, as well as major cardiovascular events including cardiovascular death, nonfatal myocardial infarction, stroke, and hospitalization for heart failure [65]. The subsequent FIGARO-DKD trial confirmed finerenone’s efficacy in reducing hospitalization for heart failure and other cardiovascular outcomes in patients with stage 1–4 CKD, including those with moderately increased albuminuria [64].

Finerenone provides benefit in heart failure primarily by reducing the risk of worsening heart failure events and cardiovascular death in patients with heart failure with mildly reduced or preserved ejection fraction (HFmrEF/HFpEF) [66,67]. The FINEARTS-HF trial demonstrated a significant reduction in the composite outcome of total worsening heart failure events and cardiovascular death (rate ratio 0.84; 95% CI, 0.74–0.95; *p* = 0.007) over a median follow-up of 32 months [66,67]. Benefits were consistent across prespecified subgroups and observed for both components of the primary endpoint. Although finerenone modestly improved patient-reported symptoms via the Kansas City Cardiomyopathy Questionnaire (KCCQ), it did not significantly impact NYHA functional class or renal composite outcomes in this population [68].

**Figure 3:**
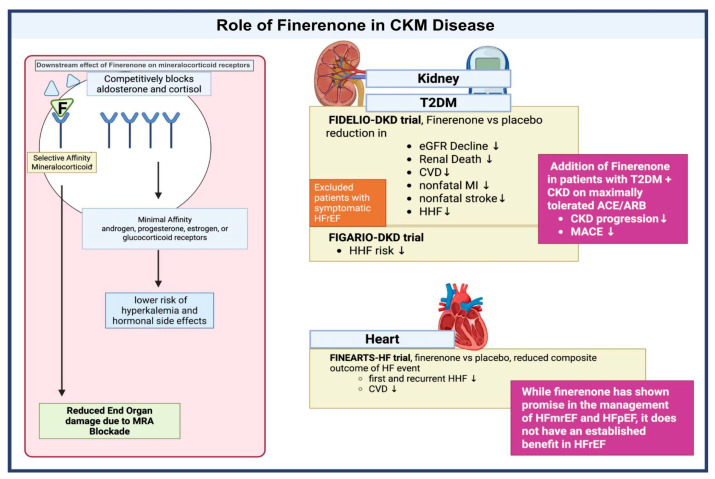
Evidence Supporting Finerenone in the Cardiovascular-Kidney-Metabolic syndrome. Legend: CKD: Chronic Kidney Disease; CVD: Cardiovascular Death; HHF: Hospitalization for Heart Failure; KCCQ: Kansas City Cardiomyopathy Questionnaire; MACE: Major adverse cardiovascular events; MRA: mineralocorticoid receptor antagonist; and T2DM: Type 2 Diabetes Mellitus.

Despite these encouraging findings, finerenone is not currently recommended as a substitute for established mineralocorticoid receptor antagonists (MRAs) in heart failure with reduced ejection fraction (HFrEF) [57]. This is not due to lack of efficacy, but rather because the FIDELIO-DKD and FIGARO-DKD trials excluded patients with symptomatic HFrEF. Importantly, the lack of evidence in HFrEF stems largely from ethical and methodological challenges in trial design [54]. Because eplerenone and spironolactone are already well-established, guideline-directed therapies in HFrEF, it would be ethically problematic to design a study where these agents are withheld in favor of finerenone, or where patients are randomized to placebo in their absence. Future studies may further elucidate finerenone’s role in HFrEF, but current evidence supports its use primarily in patients with T2DM and CKD, and in HFmrEF/HFpEF [57] [Figure 3].

Finerenone has a defined role in the management of CKM syndrome, offering targeted risk reduction in patients with type 2 diabetes, CKD, and albuminuria. When added to optimized RAS blockade and, ideally, SGLT2 inhibitor therapy, finerenone significantly lowers the risk of CKD progression and cardiovascular events, particularly heart failure hospitalizations [6]. Its inclusion in major clinical guidelines, including those from the ADA, KDIGO, and KDOQI, underscores its role as a risk-based, adjunctive therapy for patients with persistent albuminuria (UACR > 30 mg/g) despite standard care [42,69]. As evidence continues to evolve, finerenone stands as a cornerstone in bridging renal and cardiovascular protection in this high-risk population.

### 3.4. Thyroid Receptor Beta Agonists

MASLD is a significant global health concern, affecting over 30% of the adult population [70]. Characterized by the accumulation of fat in the liver due to metabolic dysfunctions such as insulin resistance (IR) and chronic low-grade inflammation, MASLD is associated with a heightened risk of cardiovascular complications [71]. In fact, CVD is the leading cause of mortality among affected individuals [71]. MASH represents a more severe form, characterized by inflammation, liver damage, and an increased risk of fibrosis, cirrhosis, and liver cancer [72]. While MASLD is not formally classified within the CKM syndrome framework, its inclusion in clinical discussions is increasingly important. MASLD shares common pathophysiologic drivers with CKM syndrome and frequently coexists with T2DM, CKD, and CVD. This overlap contributes to compounded risk and underscores the need for integrated management strategies. Recognizing MASLD in this context allows for a more comprehensive approach to risk stratification and therapeutic decision-making, particularly as emerging data continue to link hepatic steatosis with adverse renal and cardiovascular outcomes.

Management of MASLD primarily revolves around lifestyle modifications and pharmacological interventions. Among the most promising approaches are liver-specific thyroid hormone receptor β (TRβ) agonists, including GC-1 (sobetirome), KB-2115 (eprotirome), and MGL-3196 (resmetirom). Hepatocytes predominantly express TRβ, and its activation by triiodothyronine (T3) enhances key metabolic processes such as mitochondrial fatty acid uptake and β-oxidation, mitochondrial biogenesis, hepatic LDL receptor expression, and reduction in circulating LDL cholesterol [73]. These agents have demonstrated potential in reducing hepatic steatosis and improving lipid profiles. However, clinical development has faced notable hurdles: sobetirome remains in preclinical stages without phase 2 trial data, and eprotirome’s phase 3 trial was terminated due to cartilage toxicity in animal models and elevations in liver enzymes [73]. Resmetirom has emerged as the first Food and Drug Administration-approved drug for effective management of MASLD [74]. Although TRβ agonists have not demonstrated direct effects on cardiovascular or kidney endpoints, their ability to modulate hepatic metabolism and systemic cardiometabolic risk makes them relevant in the broader context of CKM syndrome.

Resmetirom is a selective TRβ agonist with 28-fold greater affinity for TRβ in the liver. This reduces the creation of new fats, increases the breakdown of fatty acids, and offers benefits against inflammation and scarring [75]. Clinical trials have demonstrated the efficacy of resmetirom in treating non-cirrhotic MASH with moderate to advanced fibrosis. In a randomized, double-blind Phase 2 trial (NCT02912260), patients with MASH (fibrosis stages F1–F3) received either resmetirom (80 mg/day, n = 78) or placebo (n = 38) for 36 weeks. Resmetirom significantly reduced hepatic fat at both 12 and 36 weeks compared with placebo [75]. Following Phase 2 results, MAESTRO-NAFLD-1 phase 3 trial (NCT04197479) was conducted. The MAESTRO-NASH trial (NCT03900429) showed that resmetirom (80 mg and 100 mg) achieved 26% and 30% NASH resolution rates, respectively, versus 10% with placebo, significantly reduced LDL cholesterol, and had mild gastrointestinal side effects. Both 52-week trials were randomized, double-blind, and placebo-controlled [74]. Younossi et al. [76]. assessed health-related quality of life (HRQL) in 125 NASH patients treated with resmetirom (n = 84) or placebo (n = 41) over 36 weeks. Resmetirom significantly improved HRQL scores compared to placebo [76].

Resmetirom demonstrated a favorable safety profile in Phase 3 trials, with mostly mild to moderate gastrointestinal symptoms [70]. Serious adverse event rates were comparable between resmetirom and placebo, and no drug-induced liver injury was reported. Cancer rates, major cardiovascular events, bone fractures, and significant BMD changes were not increased with resmetirom [70]. Resmetirom was approved in the US in 2024 for noncirrhotic NASH with moderate to advanced fibrosis, alongside diet and exercise, based on its safety profile in trials. It reduces liver fat, lowers liver enzyme levels, improves liver fibrogenesis indicators, reduces liver stiffness, and improves cardiovascular profile by lowering serum lipid levels, including LDL cholesterol [77]. Ongoing investigations are assessing the applicability of this drug in pediatric, adolescent, and adult patients diagnosed with cirrhosis. Commercially distributed as Rezdiffra, it is presented in tablet formulations of 60 mg, 80 mg, and 100 mg strengths. The prescribed daily dose is 80 mg for adult individuals weighing below 100 kg and 100 mg for those with a weight of 100 kg or greater [78].

Currently, five ongoing clinical trials are evaluating resmetirom (NCT02912260, NCT04197479, NCT03900429, NCT04951219, and NCT04643795) [79]. Further longitudinal investigations and post-marketing surveillance are essential to confirm the long-term safety of resmetirom and to detect any unforeseen off-target effects.

### 3.5. Lipoprotein-a (Lp(a))

Lipoprotein-a (Lp(a)) is increasingly recognized as a significant risk factor for premature ASCVD [80]. The AHA’s recent scientific statement highlights the causal role of elevated Lp(a) in ASCVD, a conclusion supported by extensive observational, genetic, and mechanistic evidence accumulated over decades [80]. Unlike traditional lipids, Lp(a) levels are minimally influenced by lifestyle or pharmacologic interventions and remain stable throughout life, making it a valuable biomarker for early cardiovascular risk stratification [80]. While the primary connection between elevated Lp(a) and cardiovascular disease lies in its contribution to coronary atherosclerosis, emerging data suggest potential associations with heart failure, particularly through pathways involving myocardial infarction and aortic valve stenosis [17,81]. The CASABLANCA study identified elevated Lp(a) and oxidized phospholipids as predictors of symptomatic heart failure progression, independent of coronary artery disease severity [82]. A meta-analysis of Mendelian randomization studies corroborates that higher genetically predicted Lp(a) levels are significantly associated with an increased risk of heart failure, suggesting a potential causal relationship [83]. However, current evidence does not support routine Lp(a) measurement for unexplained heart failure, and its role in the pathogenesis remains under investigation.

Notable complexities exist in the relationship between Lp(a) and cardiovascular risk. Ethnic and metabolic factors may influence Lp(a)-related risk. For example, elevated Lp(a) has shown stronger predictive value for HFpEF in White populations, suggesting possible genetic or environmental modifiers [84]. Furthermore, a more pronounced impact of elevated Lp(a) has been observed in individuals with diabetes mellitus [85]. This heightened risk in the diabetic population underscores the critical need for Lp(a) screening within the context of comprehensive cardiovascular risk assessment in these individuals. Within the CKM framework, identifying patients with both diabetes and elevated Lp(a) may enable more targeted preventive strategies.

Importantly, while statin therapy is effective in lowering LDL-C and reducing ASCVD risk, it does not reduce Lp(a) levels [86]. Data from the JUPITER trial and other studies confirm that elevated Lp(a) remains an independent risk factor even in patients achieving optimal LDL-C targets [86]. This underscores a critical unmet need for therapies specifically designed to lower Lp(a). Antisense oligonucleotides, such as APO(a)LRx, have demonstrated potent and selective Lp(a) reduction in early-phase trials, with favorable safety profiles [87]. The future of preventive cardiology and heart failure management may be significantly impacted by these Lp(a)-lowering therapies. If subsequent large-scale clinical trials confirm that lowering Lp(a) translates into reduced ASCVD events and improved survival, these agents could represent a major advancement in personalized cardiovascular prevention.

In summary, Lp(a) is a significant and potentially modifiable risk factor for premature ASCVD. While its role in heart failure remains less clearly defined, its contribution to coronary artery disease and residual cardiovascular risk is well established. Routine measurement of Lp(a) may be justified in patients with premature ASCVD, metabolic syndrome, or a family history of early-onset cardiovascular disease. As novel therapies emerge, Lp(a) may become a central target in individualized risk reduction strategies.

### 3.6. Phase 3 Trial Drugs for CKM Syndrome

Contemporary management of CKM syndrome includes lifestyle modification and established pharmacotherapies. However, a growing pipeline of novel agents, particularly RNA-based therapies and targeted biologics are expanding the therapeutic landscape by addressing previously unmodifiable risk pathways [Table 2].

RNA-based therapies, including antisense oligonucleotides (ASOs) and small interfering RNAs (siRNAs), modulate gene expression to reduce pathogenic protein synthesis. Volanesorsen, an ASO, targets hepatic APOC3 mRNA, reducing apolipoprotein C-III levels and thereby lowering plasma triglycerides [18]. While originally developed for rare conditions such as familial chylomicronemia syndrome (FCS), its mechanism is also relevant to the broader population with severe hypertriglyceridemia, a more common and clinically significant group [20,21]. Plozasiran, a siRNA targeting the same APOC3 transcript, achieves similar triglyceride-lowering effects via RNA-induced silencing complex (RISC), leading to the degradation (silencing) of the targeted mRNA [18].

Inclisiran, a siRNA targeting hepatic proprotein convertase subtilisin–kexin type 9 (PCSK9) synthesis, has completed phase III trials and is approved for LDL-C reduction in patients with ASCVD [23]. As established previously, higher levels of Lp(a) have an association with increased risk of premature ASCVD [80]. Olpasiran is a siRNA that disrupts the expression of apolipoprotein(a) gene (LPA), resulting in lower levels of apolipoprotein(a) synthesis and the final product, Lp(a). Phase III trials are currently underway to assess the effects of MACE reduction with Olpasiran in those with a history of ASCVD and Lp(a) ≥ 200 nmol/L [88]. Other agents target distinct contributors to cardiovascular risk. Ziltivekimab, a fully human monoclonal antibody against interleukin-6 (IL-6), has demonstrated reductions in high-sensitivity C-reactive protein (hsCRP), a marker of inflammation and thrombosis, in phase II trials [89]. Cholesteryl ester transfer protein (CETP) facilitates the transfer of cholesterol from high-density lipoprotein (HDL) particles to LDL and very-low-density lipoprotein (VLDL) particles, thereby increasing the concentration of the latter two. Obicetrapib is a CETP inhibitor that has been shown to lower LDL-C effectively in combination with high-intensity statins [90,91]. Angiopoietin-like 3 (ANGPTL3) inhibition through monoclonal antibodies is another therapeutic target that reduces LDL-C and triglycerides via lipoprotein lipase and endothelial lipase modulation [92]. It has demonstrated benefit in homozygous familial hypercholesterolemia independent of LDL receptor pathways.

Several RNA-based therapies are now being developed to target key components of CKM syndrome [18]. Zilebesiran, an siRNA targeting hepatic angiotensinogen, is under investigation for hypertension. Exosomal miRNAs are being explored for obesity modulation. ISIS 325568 targets insulin resistance, while CDR132L is designed to attenuate cardiac remodeling. A combination of miR-133, miR-1, miR-208, and miR-499 has shown potential in promoting cardiomyocyte regeneration. Teprasiran is being studied for acute kidney injury (AKI), and emapticap pegol targets diabetic nephropathy [18].

### 3.7. Role of Artificial Intelligence

Artificial intelligence (AI) is poised to transform the management of CKM syndrome by enabling personalized, data-driven care. Through machine learning and deep learning algorithms, AI can integrate multimodal data that includes electronic health records, laboratory values, imaging, and genomic information to identify complex patterns and predict disease trajectories. This capability is particularly valuable in CKM populations, where overlapping cardiovascular, renal, and metabolic risks complicate clinical decision-making. In patients with CKD, AI-driven models have accurately predicted cardiovascular risk by analyzing key variables such as age, comorbidities, and biochemical markers, supporting early intervention and tailored treatment strategies [93]. AI also facilitates streamlined management of interconnected factors like blood pressure, glycemic control, and nutritional status which are critical components in CKM care [93].

The American Heart Association has emphasized AI’s potential in advancing CKM management through predictive algorithms that forecast disease progression, treatment response, and complications [1]. Furthermore, AI-powered decision-support tools can assist clinicians in navigating complex treatment decisions by providing evidence-based recommendations tailored to individual patient profiles. Current applications of machine learning include early detection of acute kidney injury, identification of modifiable risk factors for CKD progression, and improved diagnostic accuracy for renal tumors [93].

Despite its promise, AI integration into clinical practice faces challenges, including data quality, model transparency, workflow compatibility, and regulatory oversight [94,95]. Addressing these barriers will be essential to ensure safe, equitable, and effective implementation. In summary, AI offers a powerful framework for enhancing diagnosis, risk stratification, and therapeutic precision in CKM syndrome. As technology evolves, its role in guiding individualized care across cardiovascular, kidney, and metabolic domains will continue to expand.

**Table 2:** The indications, mechanisms of action, outcomes, and adverse effects of phase III drugs for CKM.

Drugs	Phase 3 Trials	Principal Investigator	Indication	MOA	Outcome	Adverse Effects
Olezarsen	NCT05681351	Ionis Pharmaceuticals, Inc. [96]	Severe hypertriglyceridemia	Gal- NAc3 conjugated ASO targeting ApoC-III	Recruiting	Abdominal pain, and diarrhea.
Pelacarsen	NCT05305664	Novartis Pharmaceuticals [97]	Acute coronary syndrome (ACS), Hyperlipoproteinemia	ASO targeting Lp(a)	Pending results	Mild injection site reactions.
Plozasiran	NCT06347016	Arrowhead Pharmaceuticals [98]	Mixed dyslipidemia, Hypertriglyceridemia, Familial chylomicronemia syndrome (FCS)	siRNA targeting apoC-III mRNA	Currently recruiting	Worsening glycemic control, diarrhea, and urinary tract infection.
Inclisiran	NCT05399992	Novartis Pharmaceuticals [99]	Elevated low density lipoprotein (LDL),Atherosclerotic cardiovascular disease (ASCVD)	siRNA targeting PCSK9	Recruitment complete	Injection site reactions.
Lepodisiran	NCT06292013	Ferdinand et al. [100]	Cardiovascular disorders (CVD), Metabolic disorders	siRNA targeting ApoA	Currently recruiting	Injection site reactions, hypersensitivity reactions, and hepatobiliary adverse events.
Olpasiran	NCT05581303	UCSD Health [101]	Coronary artery disease (CAD), elevated Lp(a)	siRNA targeting Lp(a)	Pre-recruitment stage	Injection-site reactions.
Ziltivekimab	NCT05021835	Ridker et al. [102]	CVD, Chronic kidney disease (CKD)	IL-6 Blocker	Currently recruiting	Injection-site reactions.
Obicetrapib	NCT05142722	Ditmarsch et al. [103]	Heterozygous FHS, CAD	CETP Inhibitors	Completed, pending publication of results	Nausea, urinary tract infection, and headache.

## 4. Conclusions

CKM syndrome represents a complex and increasingly prevalent intersection of chronic disease, demanding a unified and multidisciplinary approach to care. Understanding CKM syndrome’s pathophysiology, risk factors, and evolving therapeutic landscape is essential for improving outcomes and reducing global disease burden. Current therapies, including SGLT2 inhibitors, GLP-1 receptor agonists, and finerenone have reshaped clinical strategies by offering integrated cardiorenal and metabolic protection. Emerging therapies targeting Lp(a), hepatic metabolism, and inflammatory pathways, alongside RNA-based technologies and AI, signal a new era of precision medicine in CKM management. Future research should focus on refining treatment strategies, optimizing multi-organ protection, and leveraging technological advancements to enhance patient care and clinical decision-making in CKM-related diseases.

## Figures and Tables

**Table 1:** Overview of selected publications defining CKM syndrome and major meta-analyses evaluating therapies relevant to CKM components (Cardiovascular Disease, Chronic Kidney Disease, and Type 2 Diabetes/Metabolic issue).

Paper Name	Authors	Year	Study Type	Population Studied	Key Findings	Inclusion Criteria/Indication	Outcome Trial Results (with *p*-Value if Available)	Side Effect Profile
Defining CKM Syndrome								
Cardiovascular-Kidney-Metabolic (CKM) syndrome: A state-of-the-art review	Sebastian S.A. et al. [3]	2024	Review	Epidemiological data from NHANES and AHA reports, highlighting prevalence across different demographics	CKM syndrome involves interconnected metabolic, cardiovascular, and renal diseases. Key mechanisms include insulin resistance, RAAS activation, oxidative stress, chronic inflammation, and lipotoxicity. The syndrome progresses through five stages, from no risk factors to symptomatic cardiovascular disease with kidney failure. Management focuses on screening, early intervention, and multidisciplinary care to reduce adverse outcomes.	N/A (Review)	N/A (Review)	1. GLP-1 RA: Primarily causes gastrointestinal issues like nausea, vomiting, and diarrhea. 2. SGLT2 inhibitors: Increase the risk of genital and urinary tract infections 3. Finerenone: May lead to hyperkalemia
Cardiovascular-Kidney-Metabolic Health: A Presidential Advisory From the American Heart Association	AHA (Ndumele C.E. et al.) [17]	2023	Presidential Advisory/Scientific Statement	General US population; focus on individuals with/at risk for CVD, CKD, T2D, Obesity.	Defines CKM syndrome as a health disorder linking obesity, diabetes, CKD, and CVD. Proposes staging (0–4) based on risk factors and disease presence. Emphasizes prevention, integrated care, and addressing social determinants of health (SDOH).	N/A (Definitional document)	N/A (Definitional document)	N/A (Recommends therapies like SGLT2i/GLP-1 RA for appropriate stages)
An Overview of Cardiovascular-Kidney-Metabolic Syndrome	Ferdinand K.C. et al. [18]	2024	Review	General overview of CKM syndrome patients.	Reinforces CKM definition, staging. Highlights role of excess/dysfunctional adipose tissue, inflammation, oxidative stress. Notes impact of SDOH and additional risk factors (chronic inflammation, family history, sleep/mental health).	N/A (Review)	N/A (Review)	N/A (Review)
SGLT2 Inhibitor Trials (Meta-Analyses)								
Dapagliflozin in Heart Failure with Mildly Reduced or Preserved Ejection Fraction	Solomon et al. [19]	2022	Phase 3, multicenter, randomized, double-blind, placebo-controlled trial	6263 patients with heart failure and left ventricular ejection fraction (LVEF) > 40%, with or without type 2 diabetes mellitus	Dapagliflozin significantly reduced the risk of worsening heart failure or cardiovascular death in patients with mildly reduced or preserved LVEF. Benefits were consistent across subgroups, including those with LVEF ≥ 60%, diabetes, and recent hospitalization.	Age ≥ 40 years, heart failure with LVEF > 40%, structural heart disease, elevated natriuretic peptides; included patients with previously reduced LVEF now >40% and those recently hospitalized	16.4% in dapagliflozin vs. 19.5% in placebo (HR 0.82; 95% CI 0.73–0.92; *p* < 0.001); Worsening HF: HR 0.79; 95% CI 0.69–0.91; Cardiovascular death: HR 0.88; 95% CI 0.74–1.05	Similar rates of serious adverse events in both groups (43.5% vs. 45.5%), no significant increase in hypoglycemia, ketoacidosis, or volume depletion; no cases of Fournier’s gangrene reported
Effects of SGLT2 inhibitors on cardiovascular outcomes in patients with stage 3/4 CKD: A meta-analysis	Li N. et al. [20]	2022	Meta-analysis	11 RCTs; 27,823 patients with stage 3/4 CKD.	SGLT2i significantly reduced primary CV outcomes (CV death/HHF) across stage 3a, 3b, and 4 CKD, irrespective of T2D or HF status.	Patients with stage 3/4 CKD included in RCTs comparing SGLT2i vs. placebo	Reduced primary CV outcome risk by 26% (HR 0.74, 95% CI 0.69–0.80, *p* < 0.001 inferred). Consistent benefit across CKD stages (*p* interaction = 0.71).	General Class Effects: Genitourinary infections, potential for volume depletion/hypotension, rare risk of DKA.
Effect of SGLT2 Inhibitors on Cardiovascular Outcomes Across Various Patient Populations	Usman, et al. [21]	2023	Meta-analysis	13 RCTs; >90,000 patients with HF, T2D, CKD or combinations.	SGLT2i consistently reduced the composite of first HHF or CV death (~23–24%) across HF, T2D, and CKD populations and combinations. Also reduced CV death (~12–16%) and HHF (~29–32%) separately.	Patients with HF, T2D, or CKD in large RCTs comparing SGLT2i vs. placebo	Reduced HHF/CV Death by ~24% (HR ~0.76–0.77, *p* < 0.001 inferred). Reduced CV Death by ~12–16% (*p* < 0.001 inferred). Reduced HHF by ~29–32% (*p* < 0.001 inferred)	General Class Effects: Genitourinary infections, potential for volume depletion/hypotension, rare risk of DKA.
GLP-1 Receptor Agonist Trials (Meta-Analyses)								
Kidney and Cardiovascular Outcomes Among Patients With CKD Receiving GLP-1 Receptor Agonists: A Systematic Review and Meta-Analysis of Randomized Trials	Chen et al. [22]	2024	Meta-analysis	12 RCTs; 17,996 participants with baseline eGFR < 60 mL/min/1.73 m^2^.	GLP-1 RAs significantly reduced composite kidney outcome, risk of >30/40/50% eGFR decline, all-cause mortality, and composite CV outcomes in patients with CKD.	Adults with varying kidney function (incl. CKD eGFR < 60) in RCTs comparing GLP-1 RA vs. control	Reduced composite kidney outcome (OR 0.85, 95% CI 0.77–0.94, *p* = 0.001). Reduced all-cause mortality (OR 0.77, 95% CI 0.60–0.98, *p* = 0.03). Reduced composite CV outcomes (OR 0.86, 95% CI 0.74–0.99, *p* = 0.03)	General Class Effects: Gastrointestinal side effects (nausea, vomiting, diarrhea), injection site reactions, rare risk of pancreatitis/thyroid tumors.
Effects of GLP-1 receptor agonists on kidney and cardiovascular disease outcomes: a meta-analysis of randomized controlled trials	Badve et al. [23]	2024	Meta-analysis (incl. SELECT trial)	11 RCTs; 85,373 participants (mostly T2D, one trial non-diabetic obesity/CVD).	GLP-1 RAs reduced composite kidney outcome, kidney failure, MACE, and all-cause death in T2D patients. Similar effects when non-diabetic SELECT trial included.	Participants (mostly T2D, one non-diabetic obesity/CVD trial) in large RCTs comparing GLP-1 RA vs. placebo	Reduced composite kidney outcome by 18% (HR 0.82, 95% CI 0.73–0.93). Reduced kidney failure by 16% (HR 0.84, 95% CI 0.72–0.99). Reduced MACE by 13% (HR 0.87, 95% CI 0.81–0.93). Reduced all-cause death by 12% (HR 0.88, 95% CI 0.83–0.93)	Higher treatment discontinuation due to AEs (RR 1.51). No difference in serious AEs vs. placebo.

## Data Availability

Not applicable.

## Abbreviations

The following abbreviations are used in this manuscript:

ACC	American College of Cardiology
ADA	American Diabetes Association
AGA	American Gastroenterology Association
AHA	American Heart Association
AI	Artificial intelligence
ACEi	Angiotensin converting enzyme inhibitor
ARB	Angiotensin receptor blocker
ASO	Antisense Oligonucleotides
ANGPTL3	Angiopoietin-like 3
ASCVD	Atherosclerotic cardiovascular disease
CAD	Coronary artery disease
CETP	Cholesteryl ester transfer protein
CKM	Cardiovascular-kidney-metabolic syndrome
CVD	Cardiovascular disease
CKD	Chronic kidney disease
EASD	European Association for the Study of Diabetes
ESC	European Society of Cardiology
GLP1-RA	Glucagon-like peptide-1 receptor agonists
HFMREF	Heart failure with mildly reduced ejection fraction
HFPEF	Heart failure with preserved ejection fraction
HFREF	Heart failure with reduced ejection fraction
KDIGO	Kidney Disease: Improving Global Outcomes
KDOQI	National Kidney Foundation Kidney Disease Outcomes Quality Initiative
LDL	Low density lipoprotein
MACE	Major adverse cardiovascular events
MASLD	Metabolic dysfunction-associated steatotic liver disease
MASH	Metabolic dysfunction associated steatohepatitis
NAFLD	Nonalcoholic fatty liver disease
NASH	Nonalcoholic steatohepatitis
PCSK9	Proprotein convertase subtilisin–kexin type 9
RAS	Renin angiotensin system
SGLT2i	Sodium-glucose cotransporter 2 inhibitors
TRβ	Thyroid receptor beta
